# Rural drinking water supply program and societal development: Evidence from the early implementation phase of India’s Jal Jeevan Mission

**DOI:** 10.1371/journal.pone.0312144

**Published:** 2024-11-21

**Authors:** Abinash Singh, Gopal Naik

**Affiliations:** 1 Indian Institute of Management Visakhapatnam, Economics and Social Sciences Area, Visakhapatnam, Andhra Pradesh, India; 2 Indian Institute of Management Bangalore, Centre for Public Policy, Bengaluru, Karnataka, India; Indian Institute of Management, Bangalore (IIMB), INDIA

## Abstract

With a community-centric approach to achieve Sustainable Development Goal 6.1, the Jal Jeevan Mission (JJM) launched in 2019, aims to improve the living standard of rural communities by addressing various issues related to rural drinking water supply system. The literature evaluating rural water supply programs in India is sparse. Emphasizing the large-scale implementation of the mission, we utilized sample survey data and multiple linear regression models to investigate whether or not access to household water tap connections has succeeded in reducing the burden of drinking water collection on women and female children in rural regions. Overall, our findings suggest moderate attainment of JJM even at a very early stage, whereas the positive achievements are reflected in terms of reducing the burden of water collection on women. Further, a higher probability of wealth-related inequity favoring advantaged groups was found in the coverage of household water tap connections both in pre- and initial-JJM period. Our study suggests ground-level strengthening of the rural drinking water supply system for achieving an equitable and sustainable outreach of the program across the country.

## 1. Introduction

**Fig 1:**
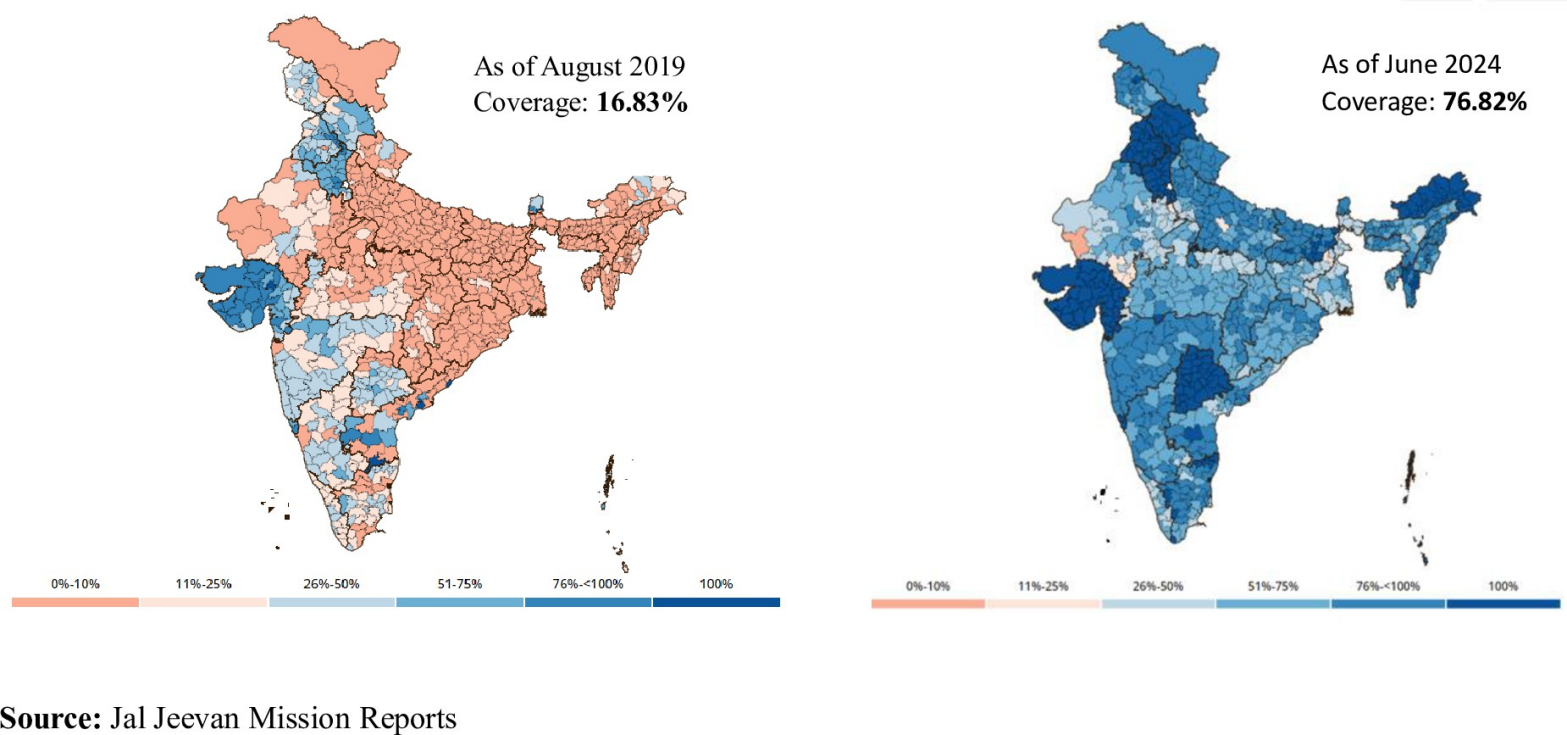
Percentage of rural households having tap water connection, 2019 and 2024.

The need for providing safe drinking water has been well recognized and given priority for rural development in India since independence. India has been making continuous efforts through public funding to provide access to drinking water, especially targeting vulnerable areas. Despite such efforts, access to safe drinking water remains a challenge in many rural regions; for instance, only 16% of rural households were having individual household tap connections in 2019 [1]. This small proportion can be ascribed to the moto of previously existing schemes, which were committed to provide access to drinking water through public drinking water tap connections installed at the village level and subsequently moved focus to habitation level access to drinking water in the recent decades [2,3]. A paradigm shift (from 16% coverage in 2019 to 76.80% as of June, 2024) in providing safe drinking water at the household level was noticed (Fig 1) after the launch of Jal Jeevan Mission (JJM)- a flagship program launched by the Government of India envisioned to provide safe, adequate and regular drinking water through individual household tap connections (Har Ghar Jal) by 2024 [4].

With the aim of achieving the Sustainable Development Goal (SDG-6) and ensuring inclusivity in access to drinking water, the mission has fast-tracked the process of providing individual tap water connection to every household with the provision of 55 litres of water per capita per day. Since its launch in 2019, when the coverage of rural households was 16.63%, the mission has managed to cover 3/4^th^ of rural households in the country. The mission aims to provide an uncompromised supply of potable water to prevent deaths and illness due to waterborne diseases, eliminate drudgery in accessing drinking water, and improve the livelihood and productivity of people in rural areas. One of the distinct features of the mission is that it focuses more on service delivery which includes sufficient, reliable, and regular supply of drinking water through household tap connections rather than simple creation of infrastructure. Moreover, with the scope of decentralized governance for monitoring the program uptake at the state (State Water And Sanitation Mission), district (District Water and Sanitation Mission) and village level (Village Water And Sanitation Committee) along with a greater community engagement, the mission holds a significant potential spillover effect in addressing health issues, lower educational attainment of girl children, lower women participation in the labour market, and also possesses the capacity to generate economic activities in various phases of its implementation. The program not only envisages access to drinking water but also aims to ensure inclusivity and equity in its coverage.

Continuous monitoring and evaluation of an intervention are two necessary conditions to assess the effectiveness of any program. Although the Government of India has come up with a platform to monitor the coverage of JJM (the IMIS database) [1], time-to-time evaluation of JJM can be crucial for policymakers to monitor the progress in the early stage. Past literature provides limelight on several spillover effects that are relatable and could be accredited to JJM upon its evaluation. For instance, easy access to safe drinking water in rural areas is reported to have transformative impact in enhancing the quality of life through improvement in health, education, gender equity, social harmony, and economic development [5–10]. A recent study unveiled the unintended impact of JJM in terms of employment generation due to the large-scale implementation, suggesting significant potential for positive externalities upon the full implementation [11]. Despite, there are hardly any program evaluation study assessing impacts of previously existing rural drinking water supply schemes in India, leaving a large scope for researchers and policymakers to understand the novelty and importance of evaluating drinking water programs. The Jal Jeevan Mission is currently under implementation, with almost 23% of rural households yet to be covered by 2024. Exploring the literature, we found sparse evidence on pre-JJM rural water supply schemes and no evidence on JJM. Highlighting upon the current implementation stage of JJM and bridging the lacuna in the literature, this study aims to draw policymakers attention towards the intermediate effects of JJM on the burden of drinking water collection on women, female children and the duration of water collection using nationally representative sample survey data.

### 1.1. Implementation phases of Jal Jeevan Mission

Jal Jeevan Mission is currently under implementation in a time-bound mission mode to ensure functional household tap connection at each rural household with adequate quantity (55 litres per capita per day) and prescribed quality (BIS:10500) on regular basis by 2024. Although JJM is a nationwide program, the states and union territories are given autonomy to implement the program based on their State Action Plan (SAP) with timelines for a 100% coverage [4]. The program launched in August 2019, started its implementation process in early 2020 following a batchwise implementation of schemes covering villages in each district. Broadly, the program aims to create water supply infrastructure in two categories: i) creation of in-village infrastructure or single village scheme (includes source development/ augmentation/ grey water management) and ii) infrastructure for bulk transfer of water or multi village scheme (includes treatment and distribution system). Creation of infrastructure for various schemes under the program may take 12 to 18 months and can be implemented in following three phases:

### Planning and mobilization phase

The planning and mobilization phases start from identification of village by DWSM (District Water and Sanitation Mission) and last till the approval of estimate for implementation of in-village infrastructure by DWSM taking approximately 3–6 months of time. This phase includes activities such as baseline mapping of existing tap connections, identification of villages, assigning department official for the implementation, community familiarization with the JJM objective, constitution of sub-committee under gram panchayat (Village Water and Sanitation Committee (VWSC) or Pani Samiti), preparation and approval of VAP (Village Action Plan) and planning for work execution.

### Implementation phase

The implementation phase takes about 6–12 months of time and includes the ground level initiation of construction based on the scheme identified. The major activities in this phase include testing of quality, augmentation of water source, creation of water supply infrastructure till household tap connections, greywater management, geo-tagging of assets, creation and maintenance of register for accounts, trial runs, installing water measurement devices (meter sensors), fixing and collection of community contribution/ water tariff.

### Post implementation phase

The post implementation phase commences after the development of water supply infrastructure and last for 3–4 months. Major activities of this phase include water supply to households, operation and management, water tariff collection and capacity building.

**Fig 2:**
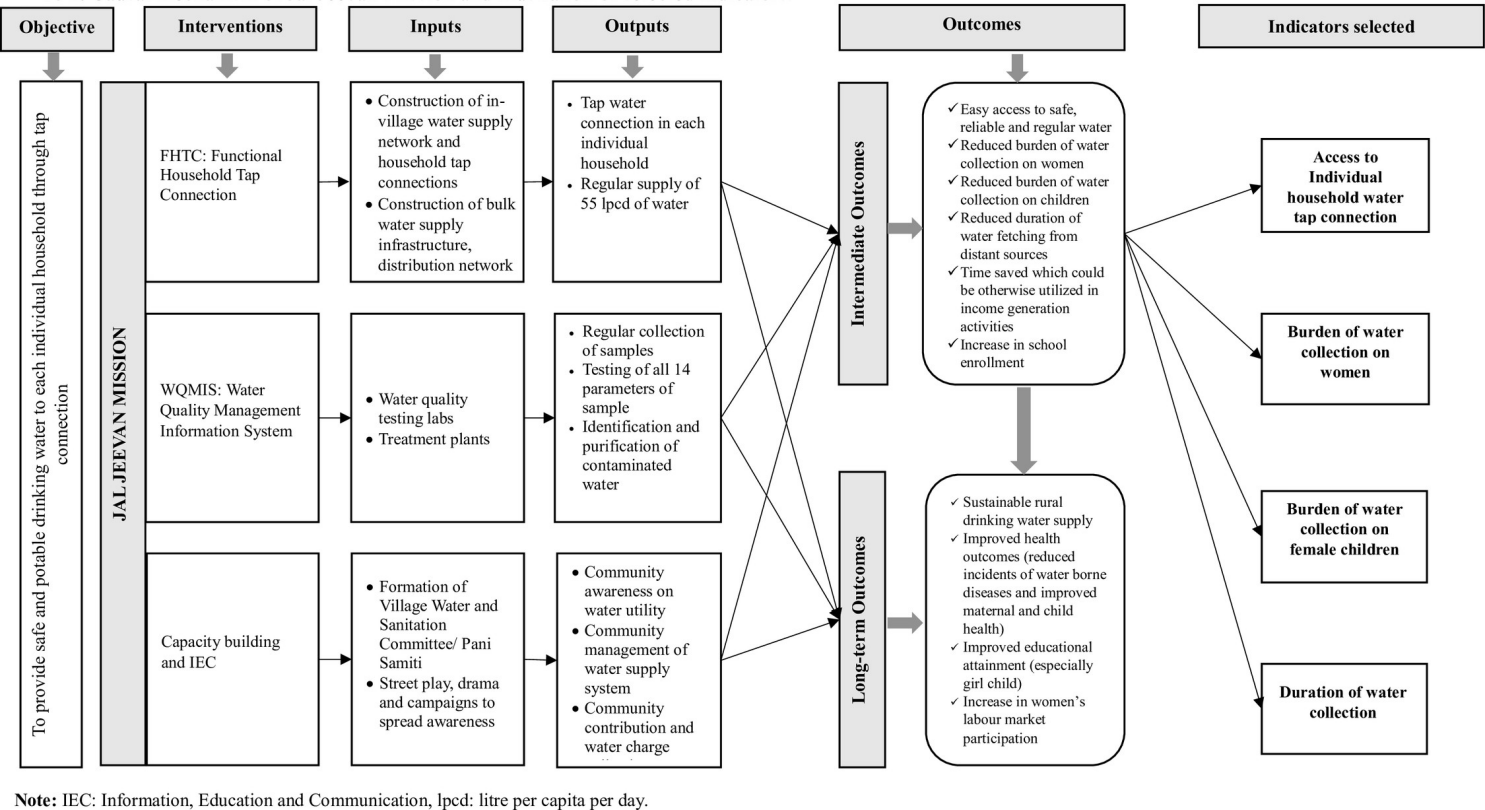
Causal mechanism of Jal Jeevan Mission and illustration of selected indicators.

Considering the time taken to develop infrastructure following various phases of implementation, 13.39% of villages were identified as ‘Har Ghar Jal (HGJ) reported’ (villages which are reported to be operational and yet to be approved by Gram Panchayat) in 2020, and this number escalated to 22.15% in 2021 [1]. However, there was no ‘HGJ certified’ villages (fully functional and approved by Gram Panchayat) during our study timeline. The first group of villages declared ‘HGJ certified’ were in 2022 which lies beyond the data collection timeline (2019–21) in our study. Hence, we anticipate the expected effect in our study to be the representation of ‘HGJ reported’ villages during 2019 and 2021. Moreover, since JJM is large program implemented across nation in a phase wise manner, the actual effect (especially in long-term) is expected to be realized only after completion of the project. Hence, the data period considered in our study aims to reflect the early/intermediate effects of JJM in terms of access to drinking water as shown in Fig 2. A detailed illustration of implementation guideline of JJM comprising of strategy for planning and implementation, institutional mechanism, implementation phases, financial planning/funding, technological interventions/innovations, support activities, water quality monitoring and surveillance, and monitoring/evaluation can be found elsewhere in the operational guidelines for the implementation of JJM [4].

## 2. Methods

### 2.1. Study design and setting

This study is a before and after cross-sectional analysis based on data from two different time periods: 2015–16 and 2019–21. We included all the Indian states in our analysis except the union territories due to the extremely low number of observations. Further, we categorized states into i) High focus Empowered Action Group States (EAG), ii) Low focus states (other than EAG and NE states), and iii) North-eastern (NE) states.

### 2.2. Data and variables

We utilized nationally representative sample survey data from round 4 (2015–16) and round 5 (2019–21) of the National Family Health Survey (NFHS). NFHS data from various rounds was accessed from the 7^th^ phase of Demographic Health Surveys (DHS). The surveys were conducted under the stewardship of the Ministry of Health and Family Welfare (MoHFW) [12] and the designated nodal agency- International Institute for Population Science (IIPS) [13]. The survey identifies a sample of villages (created considering the village population and the percentage of the population belonging to scheduled castes and scheduled tribes) as primary sampling units (PSUs) and follows a two-stage sampling method, which includes selection of PSUs using probability proportional to size (PPS) sampling in the first stage and random selection of households using systematic sampling in every selected cluster in the second stage. More detailed information on data collection techniques can be accessed from IIPS’s portal [14–17].

We considered household data sets from various rounds of NFHS and included only rural households in our study. In the fourth round of NFHS, data were collected from 601,509 households. Of these, 179,844 households belonging to urban areas and UTs were excluded, and a remainder of 421,665 households were selected as the sample in our study. The fifth round of NFHS collected data from 636,699 households, of which we excluded 164,593 households belonging to urban areas and UTs. Further, to capture the effect of JJM, we dropped 81,892 households interviewed prior to the launch of JJM (August 2019). This resulted in a final sample of 390214 households.

We selected four variables covering different aspects of access to drinking water: i) households with tap water connections in the premises, ii) adult women (age 15 to 49) in the household fetching drinking water from distant sources, iii) female children (up to age 15) in the household fetching drinking water from distant sources and iv) time elapsed in fetching water from distant sources. To estimate the program effect, the variable ‘households with tap water connections in the premises’ was used as a proxy for JJM intervention. In order to generate the above variables, we explored several drinking water-related questions that were asked to the households, such as: *What is the main source of drinking water for members of your household*?*; Where is the water source located*?*; Who usually goes to this source to fetch water for your household*?*; How long does it take to go there*, *get water*, *and come back in one trip*?.

### 2.3. Analytical tools

We employed multiple analytical tools to assess the inter-state variation, determinants, and district-level effect of access to tap water connection. We started with the calculation of state-level proportions of each of our components covering each state and state group and presented the estimates using graphs and descriptive statistics. Thereafter, we estimated a set of probit models to identify the determining socioeconomic and demographic factors in the access to drinking water. The outcome is represented by a latent variable $\left(Y_{h}^{*}\right)$, explained by a vector of explanatory variables (*X*_*h*_) such as wealth quintile, highest education level in the family, caste, religion, age of the family head, household size, and state-level fixed effect, which is expressed in the following form:

$$\begin{array}{l} Y_{h}^{*}=\beta_{0}+\beta_{1}.\;wealth\_quintile_{h}+\beta_{2}.\;fam\_education_{h}+\beta_{3}.\;caste_{h}+\beta_{4}.\;religion_{h}\\ +\beta_{5}.\;famhead\_age_{h}+\beta_{6}.\;HHsize_{h}+\beta_{7}.\;states_{h}+\mu_{h} \end{array}$$ (1)

Here, $Y_{h}^{*}$is a latent variable representing all four variables related to accessibility of drinking water. The accessibility of drinking water in rural areas was assessed using demand side as well as supply-induced indicators. The demand side indicators include the proportion of household in which women fetching drinking water from distant sources, proportion of household in which female children fetching drinking water from distant sources, and the proportion of households spending more than 30 minutes to fetch drinking water from a distant source. Meanwhile, the supply-induced indicator of accessibility is the household tap water connection (HTC) which is attributable to JJM.

Subsequently, we employed a multi-stage analysis at the district level using a set of multiple linear regression models, where we treated household tap water connection (HTC) as an exposure variable and remaining accessibility indicators as outcome variables. First, we calculated the district-level weighted average, grouped by socioeconomic status and district, for each of our outcome variables and potential confounders identified from the probit models. Further, we matched the observations of 2015–16 with 2019–21 based on unique district identifiers. Second, the change in outcome and exposure variables were calculated by taking the percentage point absolute difference in the mean. Third, the average district-level effect of JJM was estimated using two multiple linear regression models: a change score model (takes into account change in both outcome and exposure variable) and a static model (takes into account change score of the outcome variable and a static form of exposure variable). Our first model of change score estimate takes the following form:

$$\Delta y_{d}=\beta_{0}+\beta_{1}.\;\Delta HTC_{d}+\beta_{2}.\;X_{d}+\mu_{d}$$ (2)

The second model of static estimate takes the following form:

$$\Delta y_{d}=\beta_{0}+\beta_{1}.\;HTC2019\_21_{d}+\beta_{2}.\;X_{d}+\mu_{d}$$ (3)

Δ*y*_*d*_ is the change in outcome variables between 2015–16 and 2019–21, Δ*HTC*_*d*_ represents the change in the coverage of household tap connections between 2015–16 and 2019–21, *HTC*2019_21_*d*_ represents the coverage of household tap connections in 2019–21 and *X*_*d*_ is the vector of covariates included in the models such as wealth, education and caste of the households. All the statistical analyses were performed using STATA version 17.

## 3. Results

The summary statistics of the study sample from 2015–16 and 2016–21 are presented in Table 1. Among the rural households, the average size of a family was around 05, approximately one-third of the rural households belonged to the poorest wealth quintile and had no education, and around one-fourth of the households were from the SC category. Among the state groups, the highest concentration of rural households was found in High-Focus EAG states. The summary statistics of the study sample by the state groups are presented in S1 Table.

**Table 1:** Summary statistics of the study sample.

Individual Characteristics	NFHS-IV (2015–16) (N=421665)	NFHS-V (2019–21) (N=390214)
**Age of household head (Mean, SD)**	48.49 (14.28)	49.58 (14.22)
**Household size (Mean, SD)**	4.82 (2.35)	4.60 (2.24)
**Wealth quintile**		
Poorest	29.94	28.87
Poorer	26.88	26.08
Middle	21.33	21.90
Richer	13.88	15.29
Richest	07.94	07.84
**Highest education level in the household**		
No education (%)	37.75	34.50
Primary (%)	20.20	20.14
Secondary (%)	36.86	39.35
Higher (%)	05.17	05.99
**Caste**		
Schedule Caste (%)	23.40	24.77
Schedule Tribe (%)	12.45	12.95
Other Backward Caste (%)	43.71	44.79
Others (%)	20.42	17.47
**Religion**		
Hindu (%)	83.66	84.59
Muslim (%)	10.59	10.14
Christian (%)	2.50	02.11
Sikh (%)	1.71	02.03
Others (%)	1.52	01.11
**States**		
High-focus EAG states	64.83	63.96
Low focus states	24.70	26.30
North-eastern states	10.45	09.73

**Note:** Author’s estimation from the National Family Health Survey-4(2015–16) & 5(2019–21), SD: Standard Deviation, EAG: Empowered Action Group.

### 3.1. Interstate heterogeneity in access to drinking water

**Fig 3:**
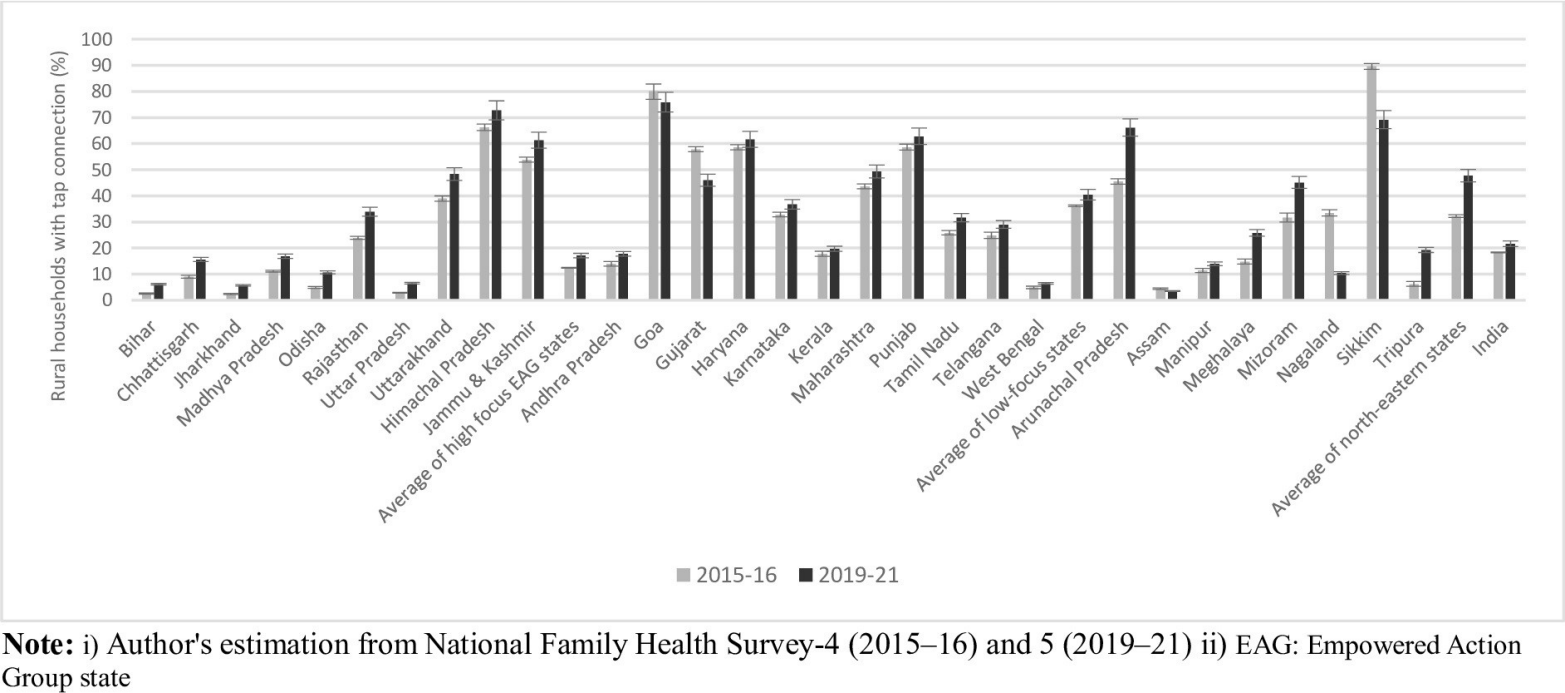
Proportion of rural households having piped drinking water facilities in their household premises, by Indian states, 2015–16 and 2019–21.

The proportion of rural households having tap water connections in their premises is presented in Fig 3. At the national level, the proportion of households with tap water connections increased to 21.62% in 2019–21 from 18.33% in 2015–16. Much of this increment was noticed among the north-eastern states (from 32.22% in 2015–16 to 47.72% in 2019–21) followed by high-focus EAG states (12.41% in 2015–16 to 17.11% in 2019–21). This increase in household level tap water connection was evident for most of the individual states except for a few states like Goa, Gujarat, Assam, Nagaland, and Sikkim, where the data indicates that the proportion of rural households with tap water connection declined over time.

**Fig 4:**
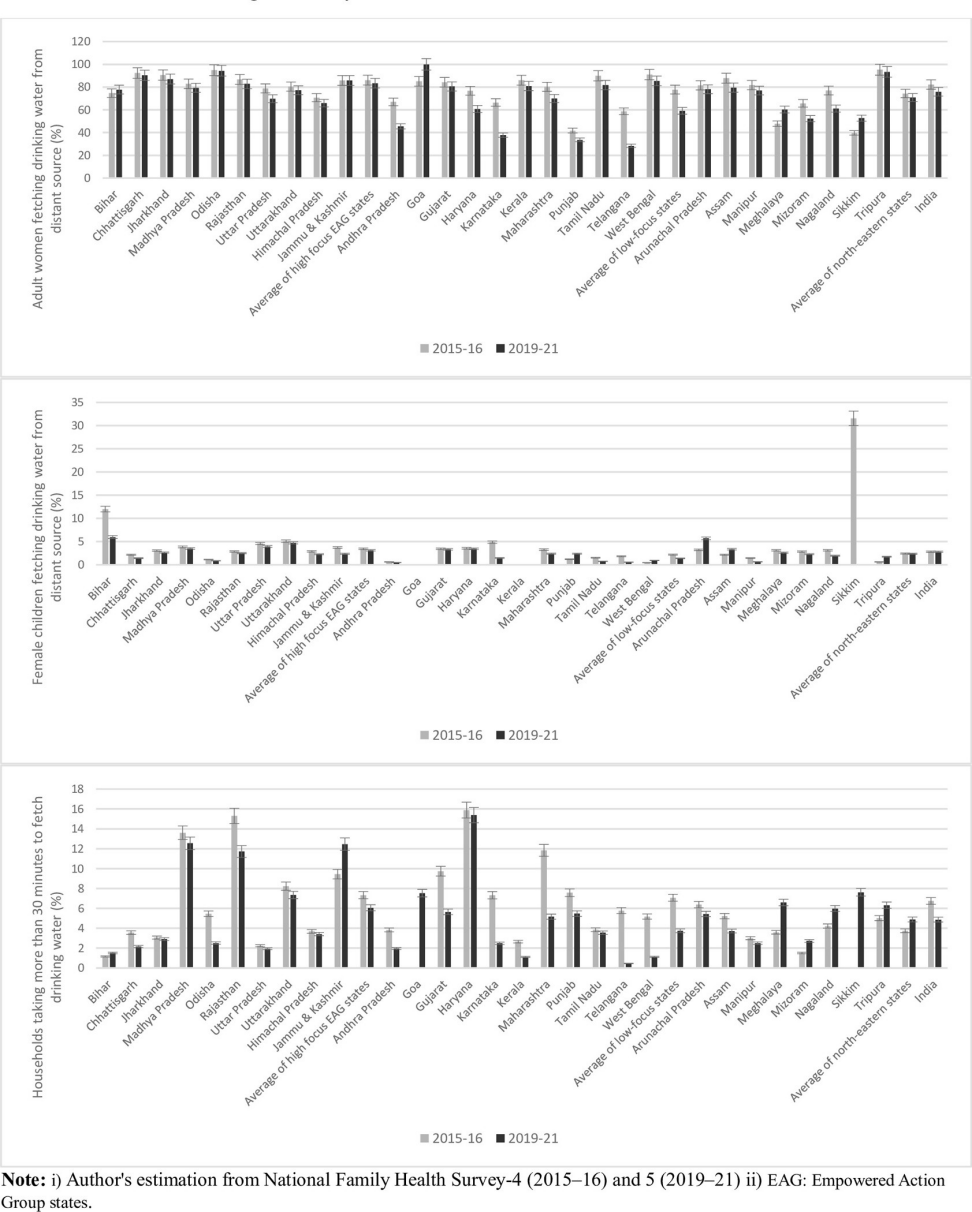
Proportion of households in which adult women and female children fetched drinking water from distant sources, by Indian states, 2015–16 and 2019–21.

Fig 4 shows the proportion of households at national and state level in which drinking water was fetched from a distant source by adult women (between age 15 to 45), female children (up to age 15), and households which took more than 30 minutes to fetch drinking water.

The proportion of adult women fetching drinking water declined at the national level from 82.25% in 2015–16 to 75.89% in 2019–21. This decline was mostly observed among the low focus states (77.77% in 2015–16 to 59.11% in 2019–21), followed by the high focus EAG states (86.13 in 2015–16 to 83.43% in 2019–21). A similar pattern of decline was noticed for the majority of the individual states except for states like Bihar, Goa, Meghalaya, and Sikkim.

Similarly, the proportion of female children fetching drinking water from distant sources decreased but marginally at the national level from 2.82% in 2015–16 to 2.81% in 2019–21. This decline was majorly evident in low focus states (2.19% in 2015–16 to 1.39% in 2019–21) followed by high focus states (3.43% in 2015–16 to 3.13% in 2019–21). The same pattern of decline was also observed at individual state level except for states like Tripura, Assam, Arunachal Pradesh and West Bengal.

Apart from the person fetching drinking water, the time taken to fetch drinking water was also found to have declined significantly at the national and majority of state levels except for selected north-eastern states. At the national level, the proportion of households taking more than 30 minutes of time to fetch water reduced from 6.75% in 2015–16 to 4.87% in 2019–21. Similarly, this reduction in time consumption was observed significantly among the majority of high-focus EAG and low-focus states; however, among north-eastern states, the proportion of households taking more than 30 minutes to fetch drinking water increased from 3.75% in 2015–16 to 4.88% in 2019–21. At the individual state level, a similar pattern of reduction time consumption to fetch drinking water was evident except for Bihar, Jammu and Kashmir, and a few north-eastern states such as Meghalaya, Mizoram, Nagaland, Sikkim, and Tripura.

### 3.2. Factors determining coverage of household tap water connections

Our supply-induced and demand-side drinking water accessibility parameters can be determined by various socioeconomic and demographic factors. Table 2 shows the marginal effect from the probit models representing the probability of having household tap connection, water collection by adult women and water collection by female children, and time taken to fetch water among various socioeconomic and demographic groups.

**Table 2:** Probability of having household tap connections (HTC), women and female children collecting water, and time taken to collect water by socioeconomic characteristics ‐ marginal effects from the probit model, 2015–16 and 2019–21.

	HTC		Water collected by women		Water collected by female children		Time elapsed for fetching water (more than 30 min)	
	2015–16	2019–21	2015–16	2019–21	2015–16	2019–21	2015–16	2019–21
Wealth quintiles **(Ref: Poorest)**								
**Poorer**	0.052*** (0.001)	0.076*** (0.001)	0.0004 (0.002)	-0.005 (0.003)	-0.003*** (0.001)	-0.002 (0.001)	-0.018*** (0.001)	-0.008*** (0.001)
**Middle**	0.113*** (0.002)	0.131*** (0.002)	-0.026*** (0.003)	-0.057*** (0.005)	-0.007*** (0.001)	-0.004*** (0.001)	-0.025*** (0.002)	-0.010*** (0.002)
**Richer**	0.187*** (0.002)	0.185*** (0.003)	-0.0106*** (0.006)	-0.165*** (0.007)	-0.010*** (0.001)	-0.011*** (0.009)	-0.035*** (0.002)	-0.018*** (0.002)
**Richest**	0.259*** (0.004)	0.260*** (0.004)	-0.0227*** (0.012)	-0.257*** (0.014)	-0.017*** (0.002)	-0.016*** (0.0002)	-0.044*** (0.003)	-0.025*** (0.002)
Family education level **(Ref: No school)**								
**Primary**	-0.004** (0.002)	-0.010*** (0.002)	-0.020*** (0.003)	-0.029*** (0.004)	-0.003*** (0.001)	-0.002 (0.001)	-0.003 (0.001)	-0.005*** (0.001)
**Secondary**	-0.011*** (0.001)	-0.020*** (0.002)	-0.032*** (0.003)	-0.043*** (0.004)	-0.002** (0.001)	-0.003*** (0.001)	-0.006*** (0.001)	-0.006*** (0.001)
**Higher**	-0.011*** (0.003)	-0.031*** (0.003)	-0.077*** (0.008)	-0.084*** (0.009)	-0.0008 (0.003)	-0.010*** (0.002)	-0.011** (0.004)	-0.006 (0.003)
Caste **(Ref: SC)**								
**ST**	-0.054*** (0.002)	-0.062*** (0.002)	0.017*** (0.003)	0.031*** (0.004)	0.002** (0.001)	0.0009 (0.001)	-0.002 (0.002)	-0.009*** (0.001)
**OBC**	-0.014*** (0.002)	-0.015*** (0.002)	0.009*** (0.003)	0.002 (0.004)	-0.0006 (0.001)	-0.0003 (0.001)	0.003 (0.001)	0.002 (0.001)
**Others**	-0.022*** (0.002)	-0.019*** (0.002)	-0.033*** (0.004)	-0.025*** (0.006)	-0.001 (0.001)	-0.0009 (0.001)	0.001 (0.002)	-0.00006 (0.004)
Religion **(Ref: Hindu)**								
**Muslim**	0.009*** (0.002)	0.018*** (0.003)	-0.012** (0.005)	-0.012 (0.008)	0.007*** (0.002)	-0.0009 (0.002)	0.008** (0.003)	0.007** (0.003)
**Christian**	0.005 (0.005)	-0.008 (0.006)	-0.001 (0.009)	-0.023 (0.012)	-0.003 (0.002)	-0.003 (0.004)	0.028*** (0.008)	-0.0003 (0.004)
**Sikh**	0.023*** (0.005)	-0.015*** (0.005)	-0.090*** (0.030)	-0.062 (0.035)	0.006 (0.017)	-0.009 (0.007)	0.012 (0.014)	0.002 (0.008)
**Others**	-0.007 (0.005)	0.013 (0.008)	-0.014 (0.009)	0.009 (0.012)	0.0006 (0.002)	-0.008*** (0.002)	-0.013*** (0.004)	0.006 (0.004)
Age of the household head	0.0002*** (0.00005)	0.0001 (0.00006)	-0.001*** (0.00009)	-0.001*** (0.0001)	-0.00004 (0.00002)	-0.0001*** (0.00003)	-7.87 (0.00005)	-0.00003 (0.00004)
Household size	-0.004*** (0.003)	-0.005*** (0.0003)	0.011*** (0.0006)	0.010*** (0.0008)	0.003*** (0.0001)	0.002*** (0.0002)	0.002*** (0.0003)	0.001*** (0.0002)
***Pseudo R2***	**0.269**	**0.199**	**0.097**	**0.150**	**0.076**	**0.08**	**0.067**	**0.09**

**Note:** i) Estimates presented are the marginal effects calculated by keeping covariates at mean, ii) estimates of marginal effects are controlled for state level fixed effect ii) standard errors are presented in parentheses iii) **, and *** denotes significance level at 5% and 1%.

In both pre-JJM and early-JJM periods, the probability of availing a household tap connection was found to be significantly high among wealthy households. The probability increased with the increase of wealth status. Compared to the poorest quintile, households belonging to the richest quintile had a 25.9% (*P = 0*.*000)* higher probability of having a household tap connection in the pre-JJM period, which marginally increased to 26% (*P = 0*.*000)* in the early-JJM period. On the other hand, the probability of water collected by women and female children and time taken to fetch water were found to be significantly high among the disadvantaged households in both pre- and early-JJM period.

Among the households who had tap water connections, the households with no education had a significantly higher probability of owning a tap water connection compared to the households with some level of educational attainment. Correspondingly, a higher probability of water being fetched by women and female children and a higher probability of longer time consumption towards fetching water was found among households with no proper education.

Among the caste categorization, households belonging to SC caste had a significantly higher probability of having household tap connections compared to the ST, OBC and other castes. At the same time, a significant proportion of households belonging to the SC caste also had a higher probability of women fetching water in their households compared to the other caste categories. However, we did not find any significant association of caste with the proportions of female children fetching water and the time taken to fetch water.

Apart from the wealth, education, and caste category, the size of the household was also found to be one of the significant determinants of drinking water accessibility. The probability of having a household tap connection was found to have significantly declined with the increase in household size. However, the probability of women and female children fetching water and a longer duration of time taken to fetch water were found to have increased with the increase in household size.

The factors determining demand and supply side indicators of access to drinking water were estimated separately for high focus EAG state, low focus states and north-eastern states and the results were similar (refer to S2–S4 Tables).

### 3.3. Access to tap water connections and its effect on the burden of water collection

**Fig 5:**
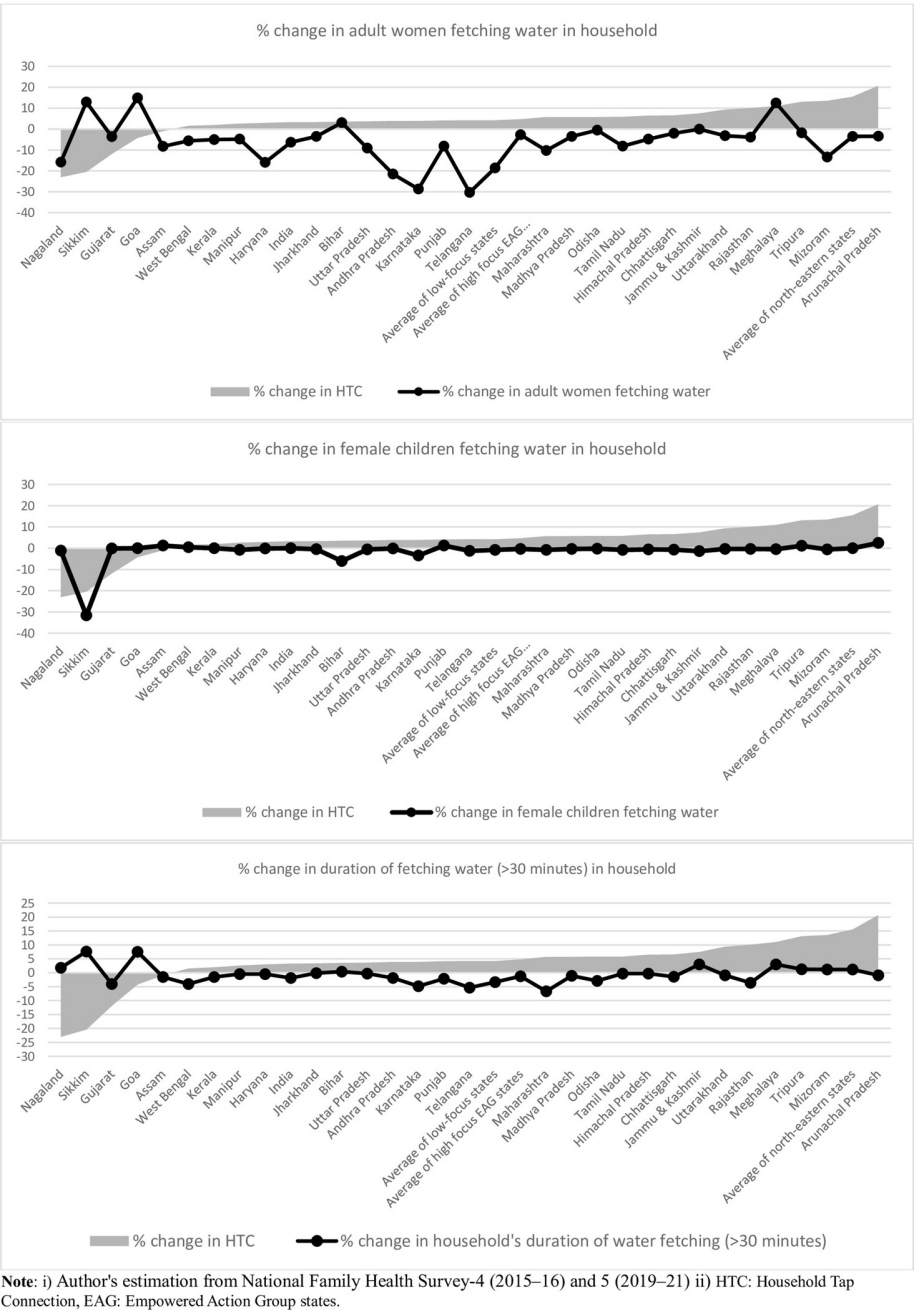
Change in proportion of households having HTC and access to drinking water, by Indian states, 2015–16 and 2019–21.

To assess the district level effect of tap water connection on the proportion of women and female children fetching water and the time taken to fetch water, we calculated the absolute difference in the mean of these outcome indicators in each state. The difference scores of all the parameters are plotted in Fig 4. A pattern of negative association between change in the proportion of household tap connections and change in accessibility parameters can be seen in Fig 5. A positive change or an increase in the household tap connection was associated with a decline in women/female children fetching water and the duration of fetching water and vice versa. However, to examine the significance of this association, we utilized regression models.

**Fig 6:**
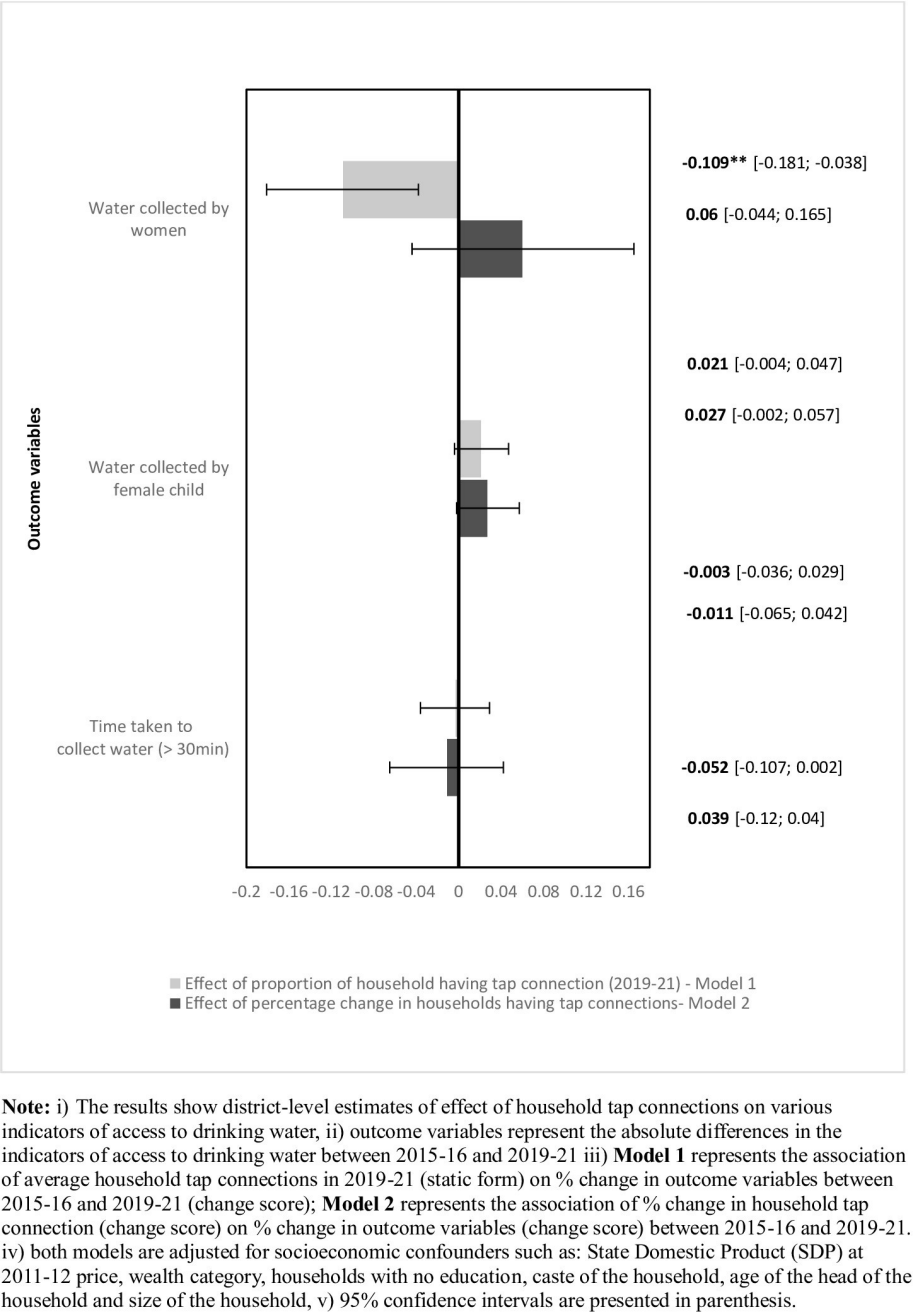
Effect of household tap connections on water collection by women and female children, time taken to collect water- district-level estimates from multiple regression analysis.

The regression coefficients from both models are plotted in Fig 6. Our result suggests the proportion of households with tap water connections in the early-JJM period had a positive effect in terms of reducing the water collection burden on adult women i.e., districts with higher household tap connections in the early-JJM period were associated with an average of 10% decline in the proportion of household in which water was fetched by adult women. We did not find any significant association between household tap water connection and reduction of burden on female children and reduction in duration of water collection.

Apart from assessing the district-level effects of JJM across the nation, we also explored various implementation phases of JJM. We identified Har Ghar Jal (HGJ) reported villages for the year 2021 as there was no HGJ-certified village as of 2021. We attempted to estimate the effect of JJM on various indicators at the district level for states with a higher proportion of HGJ-reported villages; however, we did not find any significant effect. This negligible effect could be due to the ‘HGJ reported’ status itself. It is worth noting that ‘HGJ reported’ does not necessarily confirm a regular supply of drinking water, although it can be considered as completion of water supply infrastructure development and the claim of water supply is yet to be accepted by passing a Gram Sabha resolution [1]. The state-level association of the proportion of HGJ reported villages and the demand-side water accessibility indicators are presented in S1 Fig.

## 4. Discussion

Previous studies assessing the status of rural drinking water supply systems in India were either highly skewed towards testing of water quality [18–20] or towards the socioeconomic issues and challenges faced in access to drinking water in rural areas [21–23]. Although there were countable number of studies reporting determinants of access to drinking water, community engagement, and management in rural drinking water projects [24,25], there were hardly any studies that evaluated any of the previously existing rural drinking water programs and assessed their immediate or intermediate associations with societal development. In our study, we assessed the attributable effect of JJM on burden of water collection from distant sources and duration (more than 30 minutes) of water collection. Since the program is already under implementation and every effort is made by the government to achieve the target of 100% coverage by 2024, it is difficult to assess the long-term effects of JJM on parameters such as health outcomes, education, and labour market participation at the national level. However, the intermediate effect can be assessed, which translates into the accessibility of safe and potable drinking water at the household level. Our findings suggest a moderate intermediate effect which can be attributed to JJM in its early stage of implementation.

The JJM is peculiar among the past initiatives on rural drinking water supply as it takes all the previously existing infrastructure under its umbrella and addresses several sustainability challenges by taking into account major components such as: service delivery, community ownership and management of water supply infrastructure. Besides, the JJM is a unique intervention with a proper institutional setup at the national, state, district, and village levels to supervise various components such as source sustainability, water quality, financial management, capacity building, and Information Education and Communication (IEC) [4]. The conceptualization of a rural development program such as JJM would take a considerable amount of time for the realization of its actual effect in terms of equitable and sufficient access to safe and potable drinking water along with the development of behavioral changes in the community towards the ownership and management of rural drinking water system. Our results suggest a pro-rich inequity in the coverage of household tap connections. It is important to note that this wealth-related inequity was long persistent, even before the introduction of JJM, and there was no evidence of reduction immediately after implementation of JJM began. This phenomenon of preferential biasedness associated with wealth standards is unexceptional in the implementation of large-scale public welfare programs, also commonly known as the inverse equity hypothesis [26]. Nevertheless, irrespective of the wealth-related inequity favouring higher socioeconomic groups, our findings suggest a favorable distribution of household tap connections among marginalized community especially the SC category and households with no education. Simultaneously, it was also evident that the probability of water fetching from distant sources were high among marginalized community especially the SC category and households with no education. This indicates that simple facilitation of tap water at doorstep does not encourage the consumption of water from it. Despite the effort of government in pushing the facilities towards marginalized community, people still prefer primitive source of drinking water, and this could be related to several contextual factors such as liking towards the taste and reliability of primitive sources, lack of awareness and lack of education. Further, our district-level analysis shows a positive effect of JJM on reducing the burden of water collection on women, indicating a sign of positive uptake of services provided under the JJM.

### 4.1 Policy recommendation

Despite the fact that overall proportion of households in which burden of water fetching on female children and duration of water fetching has reduced, our results could not attribute this reduction to the JJM coverage. There can be several reasons that need policy makers’ attention. First, mere creation of tap connections does not assure the flow of water as there can be several factors that can cause delay in the completion of a project in a particular village. Therefore, community involvement from the initial stage of the intervention is crucial to have a track of work progress and a regular and safe water flow. Second, community awareness on water utility development is crucial as people may still prefer the primitive source of water due to the variation in taste (chlorination) of tap water. Lastly, although the long persistence pro-rich bias in the coverage of tap water connection reduced in post-JJM period, it still exists. To tackle this issue, the government may collect community contributions based on the ability to pay and should also consider community contribution in kind. Even though the program targets a 100% inclusivity in its coverage, maintaining socioeconomic equity in its implementation stage will have a positive reflection in ensuring long-run sustainability.

### 4.2 Limitation and future scope of research

There are several limitations to our study. Our findings only indicate the early effects of JJM (till 2021) in terms of access to drinking water. This represents the initial phase of implementation in which only 22.15% of the villages were identified as ‘Har Ghar Jal reported’ and zero villages were identified as ‘Har Ghar Jal certified’ (S1 Fig). Moreover, the implementation of JJM schemes requires 12 to 18 months of time. Hence, there is a possibility that the NFHS (5^th^ round) data collected over the period 2019 to 2021 might have captured only 1^st^ and 2^nd^ phases of implementation in selected villages. Further, the true effect might not have been captured by the data due to the COVID-19 pandemic which disrupted most of the developmental activities.

As future scope of research, researchers may explore various aspects such as quantifying the time saved due to tap water connections and its utilization in income generation activities, the aspect of water quality (including quality testing and treatment), regularity and reliability of water supply system.

## 5. Conclusion

The achievement in terms of coverage of household tap connections under JJM in a very short span of time is commendable. However, drawing inferences on the effect of JJM would be imprudent as the full-implementation of the mission is yet to be achieved. Our findings are from the early implementation phase, which can be attributed to JJM and can be indicative of the mission’s final effect after its full implementation. Overall, our findings suggest a moderate effect of JJM, whereas the positive effects are reflected in terms of reducing the burden of water collection on women and achieving a caste-based equity in the coverage of household tap connections favoring the SC community. On the contrary, a rich biased distribution in the coverage of household tap water connections was found. Although JJM has expedited the overall coverage of rural drinking water supply, immediate attention of policymakers is required in prioritizing economically disadvantaged households belonging to lower wealth strata. Our study suggests ground-level strengthening of the rural drinking water supply system for an equitable and sustainable outreach of the program across the country.

## Supporting information

S1 Table Summary statistics of the study sample, by state groups. (TIF)

S2 Table Probability of having household tap connection, women and female child collecting water, and time taken to collect water in High-focus EAG states, by socioeconomic characteristics ‐ marginal effects from the probit model, 2015–16 and 2019–21. (TIF)

S3 Table Probability of having household tap connection, women and female child collecting water, and time taken to collect water in Low focus states, by socioeconomic characteristics ‐ marginal effects from the probit model, 2015–16 and 2019–21. (TIF)

S4 Table Probability of having household tap connection, women and female child collecting water, and time taken to collect water in North-eastern states, by socioeconomic characteristics ‐ marginal effects from the probit model, 2015–16 and 2019–21. (TIF)

S1 Fig State wise proportion of Har Ghar Jal reported villages, adult women fetching water, female children fetching water and duration of water fetching (>30 minutes). (TIF)
