# Supplementary figures and images for: Rural drinking water supply program and societal development: Evidence from the early implementation phase of India’s Jal Jeevan Mission
Source: PLoS One. 2024 Nov 21;19(11):e0312144. doi: 10.1371/journal.pone.0312144 (PMC11581310; doi:10.1371/journal.pone.0312144)

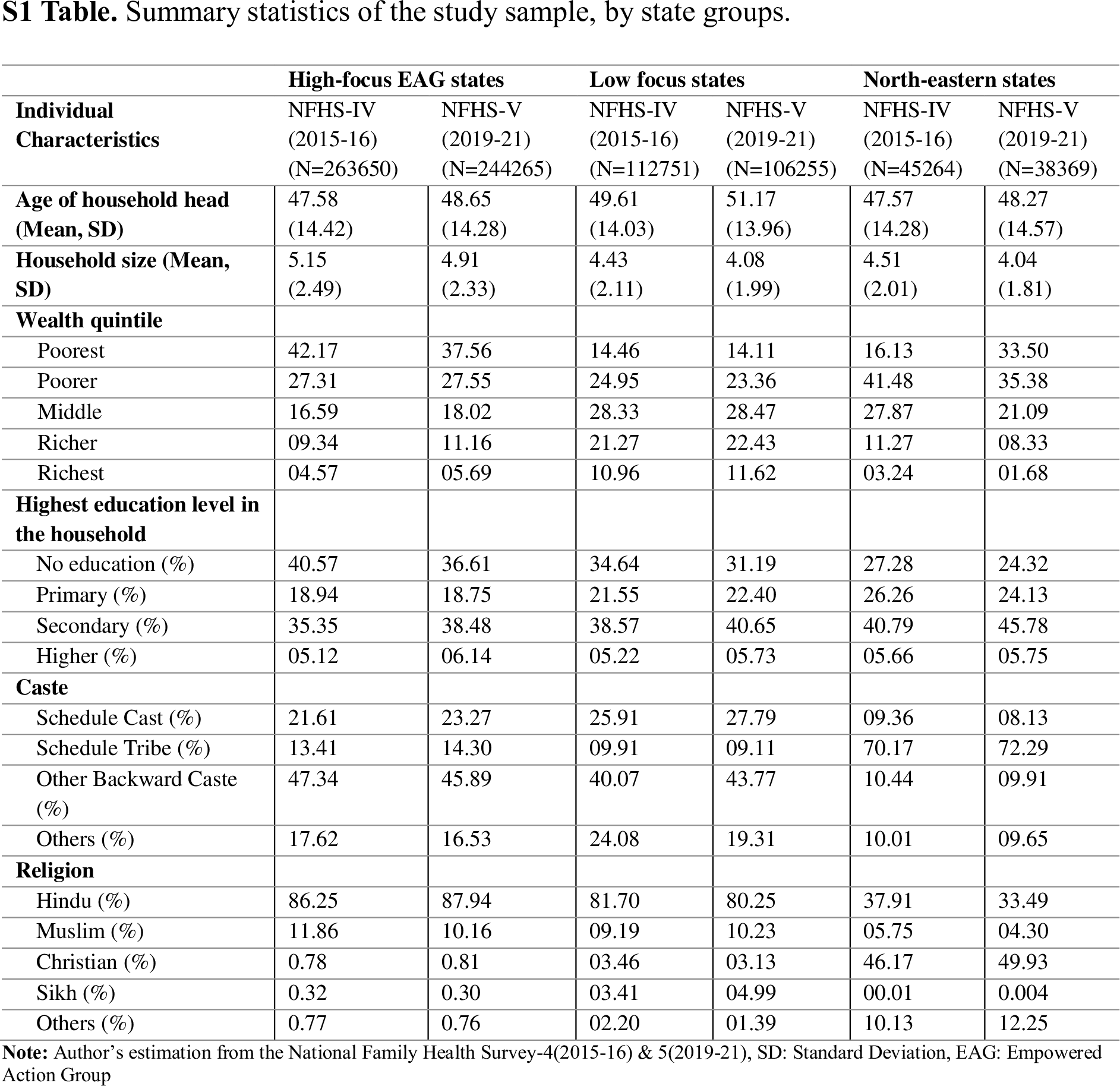

Supplement: S1 Table — (TIF) [file pone.0312144.s001.tif]

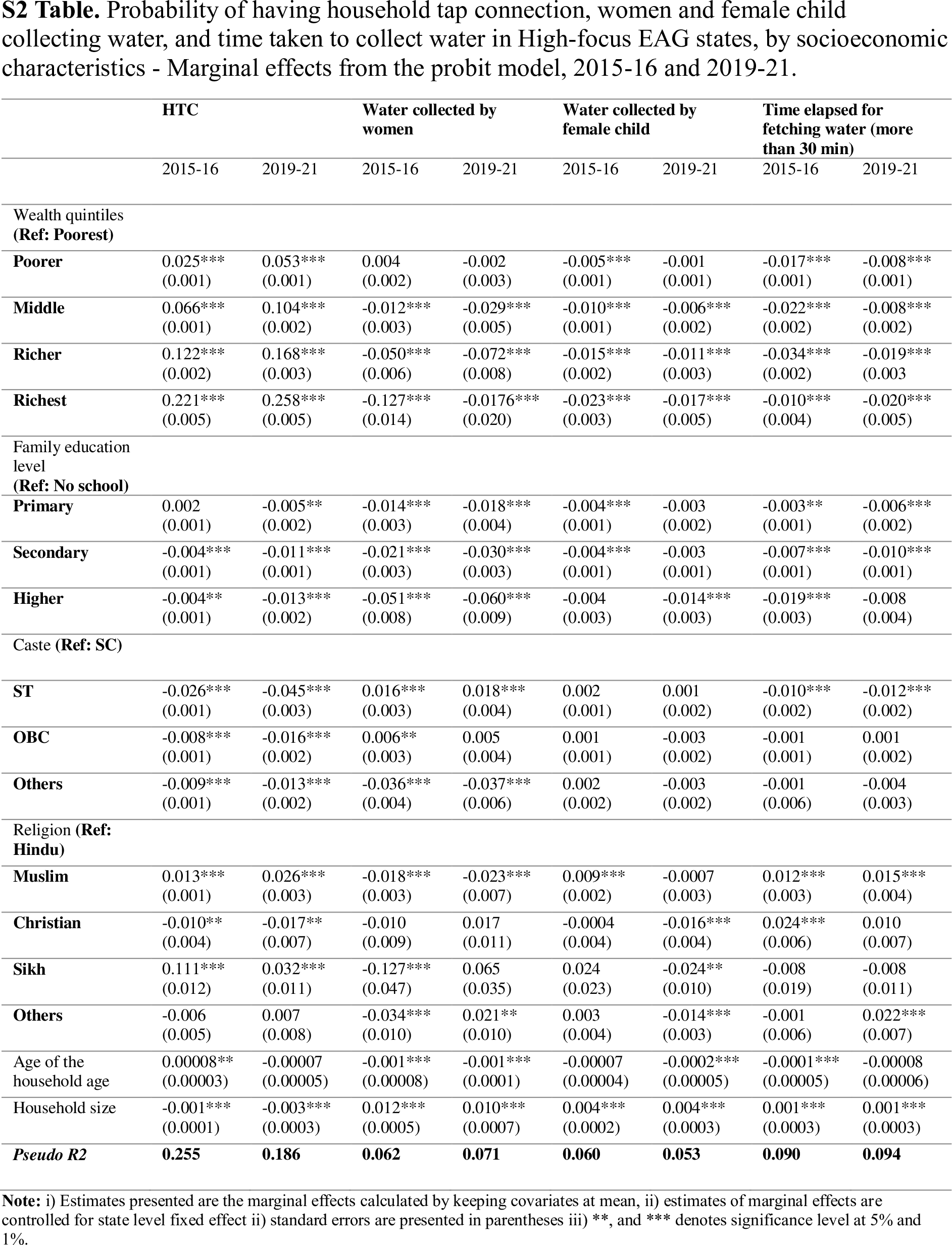

Supplement: S2 Table — (TIF) [file pone.0312144.s002.tif]

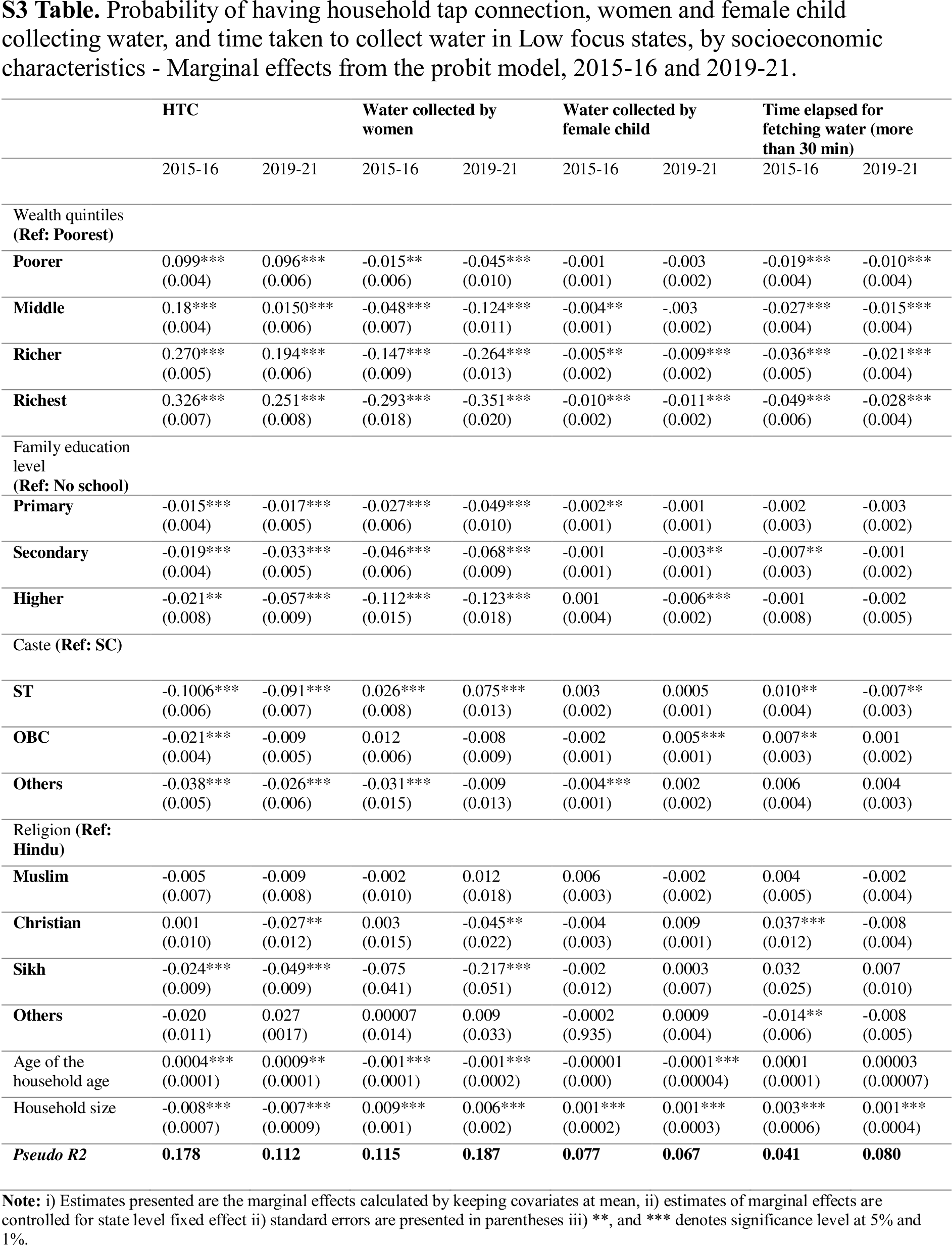

Supplement: S3 Table — (TIF) [file pone.0312144.s003.tif]

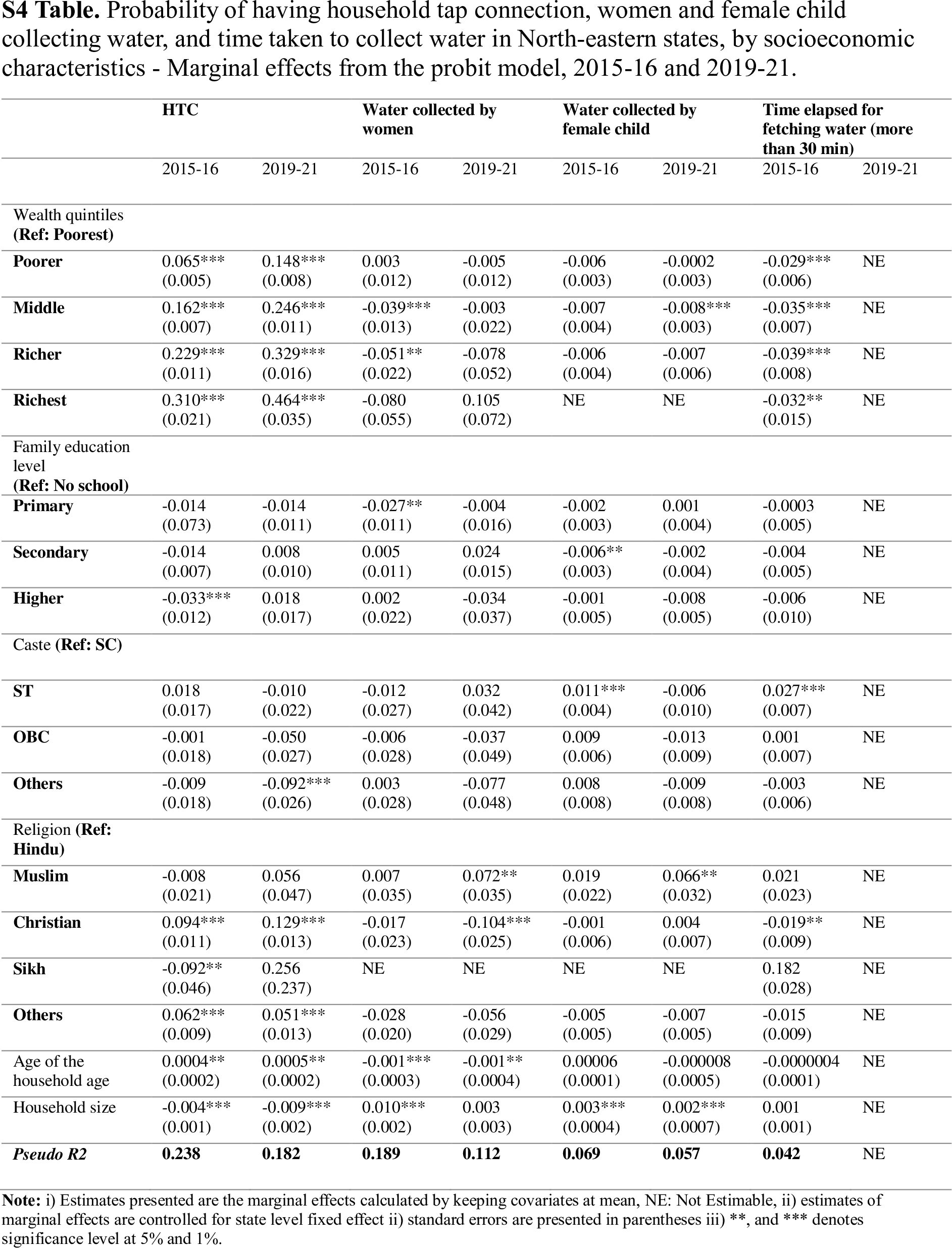

Supplement: S4 Table — (TIF) [file pone.0312144.s004.tif]

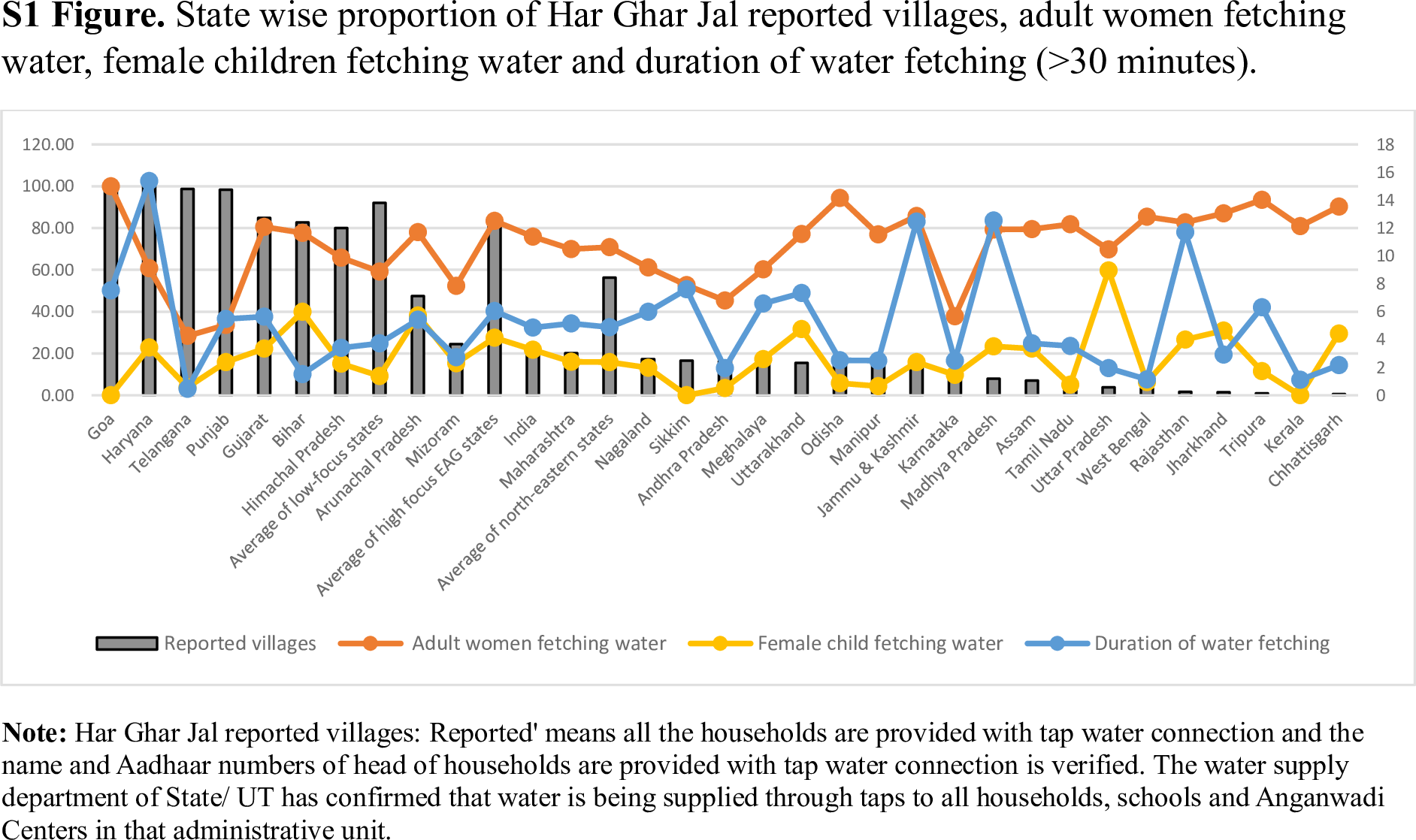

Supplement: S1 Fig — (TIF) [file pone.0312144.s005.tif]
